# Dr. Sims, on the Use of Pure Ammonia in Pregnancy

**Published:** 1799-10

**Authors:** John Sims

**Affiliations:** New Bridge Street


### *To the Editors of the Medical and Phyſical Journal.*

GENTLEMEN,

PERHAPS nothing would go further in promoting a ſucceſsful medical practice, than that every practitioner ſhould lay before the public his own knowledge of remedies which he may have long employed, with unequivocal ſucceſs, fuccefs, particularly of fuch as may not be in general ufe. Thus the late Dr. Moses Griffiths, in giving to the world his favourite chalybeate mixture, did a more effential fervice than if he had publifhed an elaborate fyftem of phyfic; and Dover's Legacy is ftill remembered for his celebrated fudorific powder, when many ingenious theories of more modern date are funk into filent oblivion.

It is with a view of throwing my mite into the medical treafury, that I fend you a prefcription which I have been in the habit of ufing for thefe fourteen years paft, and (as is well known to many apothecaries in this town) with extraordinary fuccefs, in all the complaints of pregnant women, arifing from too prevailing an acidity, fo general with them, fuch as heart-burn, vomiting, cough upon taking food, and that feverifh, reftlefs ftate fo common in the latter period of pregnancy. For all thefe complaints, I direct two or three fpoonsful of the following mixture to be taken either occafionally, or when the fymptoms are more continual, immediately after every meal:

℞ Magnefiæ uftæ, drachm. j.

Aqua puræ, unc. vfs

Spt. Cinnamomi, drachm. iij.

Aquæ Ammoniæ puræ, drachm. j.* M.

Magnefia has long been a celebrated remedy for thefe complaints, but the moft efficacious ingredient in the prefcription is the pure ammonia, as the effect will be nearly the fame without the magnefia, but this without the ammonia is far inferior indeed.

I was firft led by accident to the difcovery of the extraordinary power of the pure ammonia in correcting acidity in the ftomach, over other alkaline fubftances. My wife being feized in the night with a fevere heart-burn, I arofe with a view of getting her fome magnefia; but not being able to find any, and being defirous of procuring her fome immediate relief, I expected to obtain this by any alkaline fubftance, and not meeting with any but the water of pure ammonia, which I happened to have by me, I adminiftered twenty drops in a glafs of water; the relief was inftant, and more complete than fhe had ever experienced from taking magnefia. This induced me on another occafion to give her a tea-fpoonful of hartfhorn drops in water, expecting the fame effect; but, to my furprize, no fenfible relief was obtained, even when repeated: recourfe was had again to the pure ammonia, and with immediate fuccefs, as was afterwards found invariably to follow its ufe. This induced me to try it in others. At firft I was apprehenfive that the frequent

* This proportion fuppofes that the aq. ammon. puræ as prepared at the Apothecaries' Hall is ufed; that made by fome of the chemifts is much ftronger.

quent uſe of cauſtic volatile alkali might be attended with ſome inconvenience, and I was unwilling to believe that it could poſſeſs any power beyond any other alkaline ſubſtances, which might neutralize the acid in the ſtomach; but experience convinced me both of its ſuperior efficacy and its innocence, never having known any diſagreeable conſequences follow its uſe.

It ſhould ſeem probable from the effect of this remedy, that the cardialgia, and the other ſymptoms enumerated, may ariſe from an acid gas in the ſtomach, more than from its liquid contents. This gas is probably neutralized by the alkaline gas into which the water of pure ammonia will be converted by the heat of the body. That the carbonates of ammonia will not ſucceed, may ariſe from the ſuperior attraction of the carbonic acid for the alkali, to that of the morbid gas. But whether the theory be juſt or not, the effect is certain.

Before I conclude, it will be proper to remark, for the ſake of the younger practitioners, that the vomiting which occurs in early pregnancy, very rarely ariſes from, or is connected with acidity, and that this remedy of courſe is not appropriate. When vomiting in early pregnancy is moderate, and confined to the fore-part of the day, it appears to be uſeful, and nothing ſhould be done to prevent it; but it ſometimes happens that the vomiting is inceſſant for many days together, accompanied with great proſtration of ſtrength, and conſtant thirſt, and at the ſame time an utter inability of retaining any thing on the ſtomach. In this ſtate the moſt effectual remedy I know of is, the application of leeches to the pit of the ſtomach, and a conſtant attention to ſuffer nothing to be ſwallowed that can irritate. I have found it of the greateſt ſervice to allow the patient nothing but aſſes' milk, and that by ſingle ſpoonsful only. The uſe of leeches applied to the pit of the ſtomach in relieving vomiting is by no means confined to the ſtate of pregnancy, but when this ſymptom occurs in fevers, or follows the ingeſtion of any acrid ſtance, they are equally uſeful, as I have repeatedly experienced.

New Bridge Street,

*Sept.* 21*ſt*, 1799. JOHN SIMS.

P. S.—I have received a letter from Mr. Cook, ſurgeon, at Barking, informing me that Martha Angel, who now lives in the capacity of cook to Mr. Downing, Hatton-Garden, had the cow-pox very ſeverely, being very full, and exceedingly ill, at Highworth, in Wiltſhire, in the year 1760. Thirty years after, in the year 1790, ſhe was inoculated, and had the ſmall-pox in the uſual manner. It may be expected that I ſhould not withhold this caſe from the public; at the ſame time it muſt be acknowledged, that the experiments already inſtituted ſeem fully ſufficient to decide that the cow-pox matter which has been uſed for inoculation is effectual in preſerving the patient from any future attack of the ſmall-pox, unleſs it ſhould be true, as has has been suggested, which I deem very improbable, that the cow-pox enables the constitution to resist the contagion of the small-pox for a certain length of time only. It appears more probable that there may be different diseases among the cows, which are not very accurately distinguished; and in this point of view, the publication of this and similar cases may have its use, in exciting a due care, that the genuine disease only be taken for the purpose of inoculation.

